# Côte d’Ivoire Dual Burden of Disease (CoDuBu): Study Protocol to Investigate the Co-occurrence of Chronic Infections and Noncommunicable Diseases in Rural Settings of Epidemiological Transition

**DOI:** 10.2196/resprot.8599

**Published:** 2017-10-27

**Authors:** Ikenna C Eze, Clémence Esse, Fidèle K Bassa, Siaka Koné, Felix Acka, Loukou Yao, Medea Imboden, Fabienne N Jaeger, Christian Schindler, Mireille Dosso, Véronique Laubhouet-Koffi, Dinard Kouassi, Eliézer K N’Goran, Jürg Utzinger, Bassirou Bonfoh, Nicole Probst-Hensch

**Affiliations:** ^1^ Swiss Tropical and Public Health Institute Basel Switzerland; ^2^ University of Basel Basel Switzerland; ^3^ Centre Suisse de Recherches Scientifiques en Côte d’Ivoire Abidjan Côte d'Ivoire; ^4^ Institut d’Ethnosociologie, Université Félix Houphouët-Boigny Abidjan Côte d'Ivoire; ^5^ Unite de Formation et de Recherche Biosciences, Université Félix Houphouët-Boigny Abidjan Côte d'Ivoire; ^6^ Institut National de Santé Publique Abidjan Côte d'Ivoire; ^7^ Ligue Ivoirienne contre l’Hypertension Artérielle et les Maladies Cardiovasculaire Abidjan Côte d'Ivoire; ^8^ Institut Pasteur de Côte d’Ivoire Abidjan Côte d'Ivoire

**Keywords:** cohort study, biobanking, biomarkers, Taabo HDSS, dual disease burden, humans, hypertension, diabetes mellitus, malaria, helminths

## Abstract

**Background:**

Individual-level concomitance of infectious diseases and noncommunicable diseases (NCDs) is poorly studied, despite the reality of this dual disease burden for many low- and middle-income countries (LMICs).

**Objective:**

This study protocol describes the implementation of a cohort and biobank aiming for a better understanding of interrelation of helminth and Plasmodium infections with NCD phenotypes like metabolic syndrome, hypertension, and diabetes.

**Methods:**

A baseline cross-sectional population-based survey was conducted over one year, in the Taabo health and demographic surveillance system (HDSS) in south-central Côte d’Ivoire. We randomly identified 1020 consenting participants aged ≥18 years in three communities (Taabo-Cité, Amani-Ménou, and Tokohiri) reflecting varying stages of epidemiological transition. Participants underwent health examinations consisting of NCD phenotyping (anthropometry, blood pressure, renal function, glycemia, and lipids) and infectious disease testing (infections with soil-transmitted helminths, schistosomes, and Plasmodium). Individuals identified to have elevated blood pressure, glucose, lipids, or with infections were referred to the central/national health center for diagnostic confirmation and treatment. Aliquots of urine, stool, and venous blood were stored in a biobank for future exposome/phenome research. In-person interviews on sociodemographic attributes, risk factors for infectious diseases and NCDs, medication, vaccinations, and health care were also conducted. Appropriate statistical techniques will be applied in exploring the concomitance of infectious diseases and NCDs and their determinants. Participants’ consent for follow-up contact was obtained.

**Results:**

Key results from this baseline study, which will be published in peer-reviewed literature, will provide information on the prevalence and co-occurrence of infectious diseases, NCDs, and their risk factors. The Taabo HDSS consists of rural and somewhat more urbanized areas, allowing for comparative studies at different levels of epidemiological transition. An HDSS setting is ideal as a basis for longitudinal studies since their sustainable field work teams hold close contact with the local population.

**Conclusions:**

The collaboration between research institutions, public health organizations, health care providers, and staff from the Taabo HDSS in this study assures that the synthesized evidence will feed into health policy towards integrated infectious disease-NCD management. The preparation of health systems for the dual burden of disease is pressing in low- and middle-income countries. The established biobank will strengthen the local research capacity and offer opportunities for biomarker studies to deepen the understanding of the cross-talk between infectious diseases and NCDs.

**Trial Registration:**

International Standard Randomized Controlled Trials Number (ISRCTN): 87099939; http://www.isrctn.com/ISRCTN87099939 (Archived by WebCite at http://www.webcitation.org/6uLEs1EsX)

## Introduction

There is an alarming increase in the prevalence of noncommunicable diseases (NCDs) in low- and middle-income countries (LMICs). Although this is generally considered the result of an increase in life expectancy and westernization of lifestyle, these common and widely accepted NCD risk factors do not provide a complete picture [1]. In fact, within high-income countries, the risk of cardiovascular disease among migrants from LMICs remains elevated [2,3]. A relevant component of NCD may be attributed to infection/inflammation and neglected tropical diseases (NTDs), which, despite the epidemiological transition, remain high in LMICs, particularly in their neglected populations [4]. For Côte d’Ivoire, the 2010 Global Burden of Disease study showed both infectious diseases (IDs) and NCDs to be key drivers of morbidity and mortality [5]. On one hand, ID morbidity and mortality rates remain high, especially in the most vulnerable communities. On the other hand, there is a high prevalence of NCD risk factors, as evidenced already by the 2005 population-based World Health Organization Steps study in Abidjan and surroundings [6]. In the Taabo health and demographic surveillance system (HDSS) for routine monitoring of vital statistics in south-central Côte d’Ivoire, NCDs were the main cause in 20% of the deaths, which were mostly of cardiovascular origin, while IDs remained the primary cause of death [7].

### Inflammation and Cardiometabolic Phenotypes

Cardiovascular disease and diabetes mellitus contribute significantly to the disease burden in LMICs. They are also of particular interest with potential links to infections, as they are associated with altered immune and inflammatory responses [8]. Cardiovascular disease, diabetes, and their preclinical phenotypes (eg, atherosclerosis and metabolic syndrome) as well as their causal risk factors (eg, obesity, smoking, and air pollution) are associated with inflammation. Systemic low-grade inflammation promotes insulin resistance and atherosclerotic plaque formation. Barker formulated the hypothesis of the childhood origin of chronic age-related conditions [9], and in fact, inflammation is already associated with carotid intima media thickness and insulin resistance in children and adolescents [10]. For populations in LMICs, the link of cardiovascular disease and diabetes with inflammation implies that individuals surviving lethal IDs by mounting a strong inflammatory response may become more susceptible to age-related inflammatory phenotypes later in life [3,11-13]. In addition, repeated and chronic infections may lead to subtle cell and organ damage and permanent derailment of the immune system, which could enhance susceptibility to NCD risk factors later in life. In fact, a significant proportion of the disability-adjusted life years attributed to cardiovascular disease in LMICs is related to inflammation precursors, such as rheumatic fever and other NTDs [4]. The role of infections as risk factors for diabetes remains poorly understood [14] even though it is thought that diabetes puts patients at higher risk for infections that are prevalent in LMICs [8,15]. Thus, there is an overall lack of adequate data to determine the true extent of cardiovascular disease and diabetes that result from or are prevented by infections in LMICs.

### Helminth Infections and Cardiometabolic Phenotypes

Helminth infections contribute substantially to the NTD-associated global burden of disease, and several are highly prevalent in many parts of the world [16]. Soil-transmitted helminths and schistosomes are among the most prevalent infections in human populations in LMICs [17]. The contribution of helminths to NCD is the focus of intense research [18-21]. Individuals with heavy and chronic helminthic infections can suffer from malnutrition, stunted growth, anemia, and cognitive impairments, which may have a direct impact on NCD risk or an indirect impact through lifestyle measures [22]. Several animal models show a beneficial influence of helminth infections on metabolic homeostasis [19,23]. Cross-sectional epidemiological studies showed an inverse relationship between diabetes and lymphatic filariasis [24,25]. Infection with soil-transmitted helminths was associated with lower insulin resistance and lipid levels [26]. While studies on schistosomiases and strongyloidiasis reported negative association with glucose tolerance [27,28], other studies reported a beneficial effect, and even lower prevalence of diabetes in association with *Schistosoma japonicum* [29]. A study of Australian Aboriginal adults revealed a strong inverse relationship between infection with *Strongyloides stercoralis* and type 2 diabetes [30]. Epidemiological evidence on helminths and hypertension is very limited. A study among schoolchildren in Uganda found helminth infections to be positively associated with blood pressure [31]. Generally, helminth infections are clinically asymptomatic, but their direct and indirect effects on the host’s immune system might be of particular relevance to the host’s susceptibility to NCDs. The NCD risk may further depend on the host-pathogen relationship based on their co-evolved genetic variation. Endemic helminth infections likely exerted a strong selective pressure contributing to specific genetic host factors conferring an altered risk for immune-mediated diseases [32].

### Malaria and Cardiometabolic Phenotypes

Malaria still ranks highest on the Global Burden of Disease list for many African countries, including Côte d’Ivoire [5]. In the first 3 years of running the Taabo HDSS, malaria contributed to 20% of all registered deaths, ranking as the number one cause of death [7,33]. Although fever, a common symptom of malaria, can go along with stress-induced hyperglycemia [34,35], there is a lack of epidemiological evidence on the association of malaria with parameters of the metabolic syndrome or diabetes. According to clinical studies, malaria, even in the uncomplicated form, led to altered lipid profiles, which in some cases were prolonged, despite malaria treatment [36]. The malaria-related changes in lipid profiles have not been studied for their potential role in atherosclerosis and hypertension. A higher prevalence of *Plasmodium falciparum* infection was found among persons with diabetes [37]. In addition, fasting glucose, glycosylated hemoglobin (HbA1c), and insulin resistance were higher among non-diabetic persons with malaria [38]. Maternal malaria has also been demonstrated to have an impact on blood pressure and glucose in the offspring, thereby increasing the risk of developing hypertension and diabetes later in life [9]. Recent evidence from the African Genome Variation Project demonstrated that genetic loci under positive selection in individuals of African compared to European descent were related to malaria susceptibility on one hand (*CR1*) and osmoregulation and hypertension (*ATP1A1* and *AQP2*) on the other hand [39]. Interestingly, preliminary evidence suggests that variation in *CR1* may be related to coronary artery disease and hypertension [40]. Individuals of African descent have higher levels of circulating angiotensin-II due to its protective effects on malaria, thus genetic positive selection of the variants in the renin-angiotensin-aldosterone system may have been driven by malaria [41]. African-Americans have a higher prevalence of hypertension than Caucasians living in the same settings [42]. Hence, it has been hypothesized that persons with ethnic origins from malaria-endemic regions may be more susceptible to hypertension (due to co-evolution and positive selection of protective genetic variants).

### Relevance Health and Demographic Surveillance System With Integrated Biobanks

A complex array of different infection, environment, host lifestyle, and genome interactions are expected to drive immunological and inflammatory diseases, necessitating individual-level complex data to disentangle these disease networks. Hence, there is a pressing need to better understand the links between IDs and NCDs, to enable the development of integrated approaches for intervention against this dual disease burden. In LMICs where vital statistics and setting-specific health information are scarce, HDSS is recognized as a powerful platform for assessing and longitudinally monitoring key demographic parameters and disease burden [43,44]. Moreover, HDSS provides a unique means for population-based epidemiological and health systems research, and evidence collected in the context of HDSS effectively feeds into policy [44,45].

**Figure 1:**
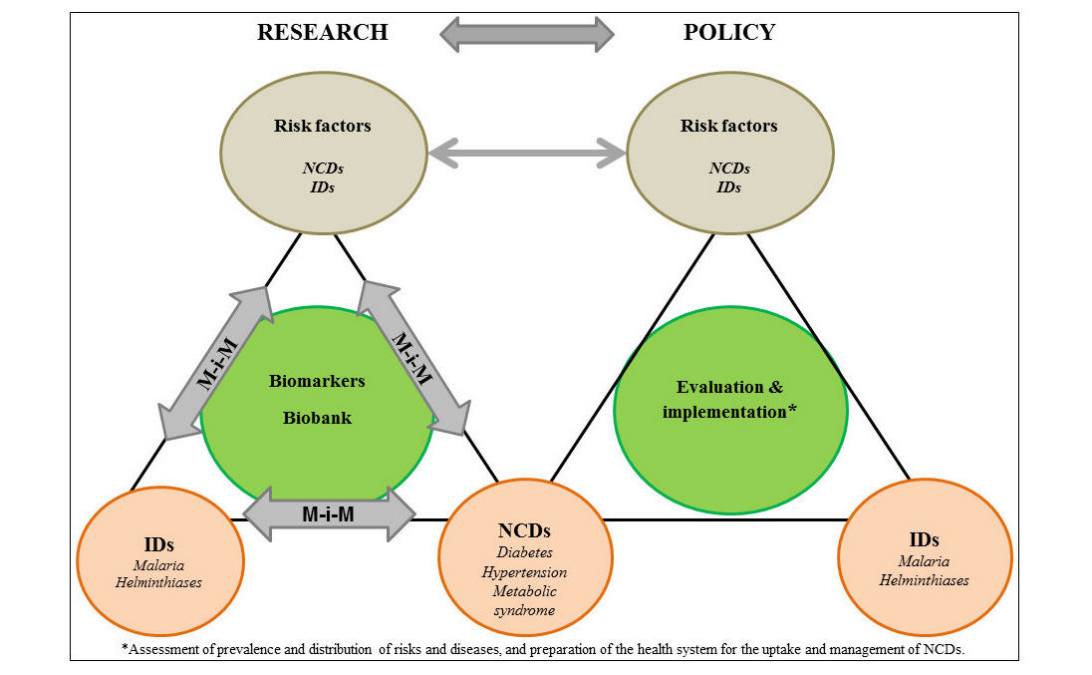
Framework of the CoDuBu study (IDs: infectious diseases; NCDs: noncommunicable diseases; M-i-M: meet-in-the-middle paradigm).

The integration of biomarkers into research protocols are of relevance to assign causality to observed associations, improve mechanistic understanding of diseases, and identify novel targets for disease screening, diagnosis, treatment, and surveillance. The availability of broad “-omics” analyses of biospecimens allows for systemic approaches (including systems medicine and exposome research) that will strengthen the investigation of disease mechanisms and considerably improve the understanding of disease mechanisms [46,47]. Applying the “meet-in-the-middle paradigm”, linking biomarkers to both exposures and health outcomes—or in the case of this project to both IDs and NCDs—will allow the investigation of biomarkers likely to play a role in the causal pathway from exposure to disease (Figure 1). Therefore, this methodological approach of meet-in-the-middle is of potential relevance for identifying preventive and therapeutic targets [48,49]. Venous blood (suitable for cytokine profile, DNA extraction, and subsequent methylation profile typing) [50], urine (suitable for metabolomics profile detection) [51], and stool samples (suitable for gut microbiome typing) [52,53] constitute common biospecimens applied towards biomarker assays in disease mechanistic linkage. Novel protocols for biospecimen sampling, processing, and storage are essential for the establishment of high-quality biobanks in remote areas of LMICs in the context of dual disease burden research.

### Objectives of the CoDuBu Study

The overarching goal of this project is to deepen our understanding of the dual burden of IDs (helminthiases and malaria) and NCDs (hypertension and diabetes) among adults in the Taabo HDSS in south-central Côte d’Ivoire, to set the stage for future mechanistic research, and to translate key findings into policy action. We will pursue the following specific objectives:

1. To conduct a population-based cross-sectional survey in adults from rural and semi-urban parts of the Taabo subdistrict to assess (a) the prevalence, intensity, and distribution of helminth (*Ascaris lumbricoides*, *S. stercoralis*, hookworm, *Trichuris trichiura*, *Schistosoma haematobium,* and *Schistosoma mansoni*) and *Plasmodium* infections, (b) the prevalence and distribution of metabolic syndrome, hypertension, and diabetes, and (c) the co-occurrence of these IDs and NCDs and the distribution of these comorbidities and their respective risk factors at the level of individuals.
2. To establish a biobank consisting of blood, urine, and stool as an investment for future longitudinal biomarker research on the mechanistic link between helminth and *Plasmodium* infections, and hypertension and diabetes.
3. To prepare the local health system for the uptake and management of NCDs through assessment of deficiencies and provision of necessary training, instruments, and facilities to the local health staff and health centers.

## Methods

### Location

**Figure 2:**
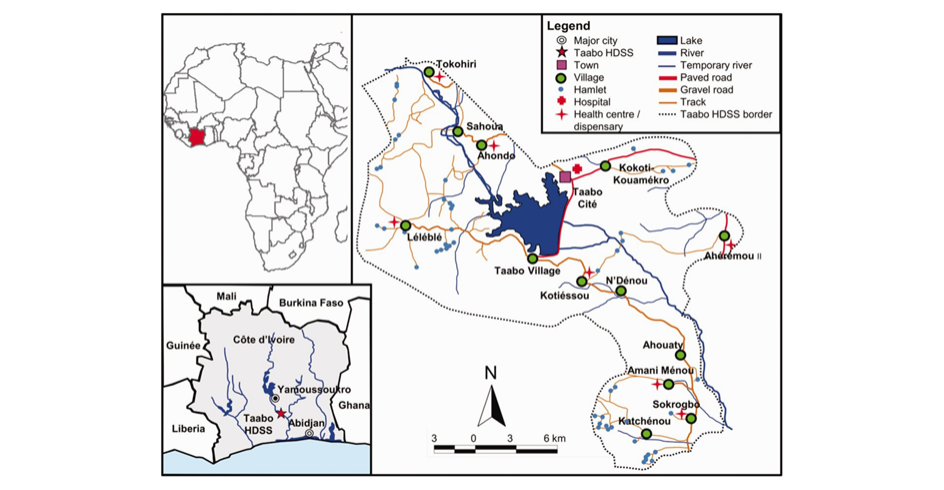
Map of the Taabo HDSS, located in south-central Côte d’Ivoire. The sites of the CoDuBu baseline study were Taabo-Cite, Tokohiri (36 km north of Taabo-Cite) and Amani-Menou (40 km south of Taabo-Cite). (Figure is reused with permission from Oxford Academic Journals [33]).

The study was conducted in the Taabo HDSS, which is located about 150 km north-west of Abidjan, in the Agneby-Tiassa region of south-central Côte d’Ivoire. The Taabo HDSS covers a surface area of about 980 km^2^and includes an urban setting (Taabo-Cité), 13 villages, and more than 100 small hamlets [33]. The Taabo HDSS was set up in 2008 and has been operational since 2009. It collects longitudinal demographic data at the individual and household level, usually done in three rounds per year [33,54]. The 2012 demographic information of the Taabo HDSS has been published elsewhere [33]. In brief, crude birth and mortality rates were 33.9 and 8.2 per 1000 population. In- and out-migration rates were 136.9 and 160.1 per 1000 population respectively, with a population growth rate of 2.5 per 1000 population. Male to female ratio was 104:100, and total fertility rate was 4.8 children per woman. Life expectancy was 61 years for males and 65 years for females. Main disease patterns include malaria and NTDs [33]. Deaths are usually reported by key informants, and verbal autopsies are conducted using standard protocols to determine causes of death [7,33]. Main causes of death include malaria, HIV/AIDS, and tuberculosis [7]. Specific in-depth questionnaire and epidemiological surveys have previously been carried out on subsamples of the population to deepen the understanding of malaria, NTDs, and iron-deficiency anemia [54-58]. The HDSS population (approximately 42,000 individuals from 6700 households) is predominantly Ivorian, with Akan as the main ethnic group. There are eight health facilities in the Taabo HDSS area: a 12-bed hospital in Taabo-Cité and seven health centers and dispensaries across the 13 villages [33]. Figure 2 shows a map of the Taabo HDSS.

We selected three areas from the Taabo HDSS for the purposes of this study. Selection was based on degree of urbanization, potential to provide at least one adult per household towards reaching an effective sample size, prevalence of malaria and helminthiases from preliminary surveys, availability of health center for examination, proximity to the main hospital in Taabo-Cité for ease of referral, and higher potential for follow-up. We therefore selected Taabo-Cité, Amani-Ménou (40 km from Taabo-Cité in the south) and Tokohiri (36 km from Taabo-Cité in the north) reflecting semi-urban, rural, and very rural settings in the Taabo HDSS.

### Preparation

For site preparation, the study team visited the district and village chiefs and informed them about the study. In addition, the hospital and health centers in the selected sites were visited for assessment of diagnostic and treatment capabilities for hypertension and diabetes and for malaria and helminthiases. Informing the local population about the study included information events planned by the HDSS staff, in collaboration with the CoDuBu study team. The HDSS field staff had 2-day training on performing interviews and instruction of participants on biospecimen collection. The medical team also underwent 2-day training on health examinations and laboratory screening. The study procedures were piloted on 10 local residents, who would not be participating in the main study.

### Design

This was a population-based cross-sectional study among adult participants aged ≥18 years, randomly sampled within the selected Taabo HDSS areas. The examinations and interviews were conducted between April and August 2017. A biobank was established for future biomarker research based on biospecimens collected from the participants. The survey occurred in two phases.

Phase I (health examination; April and May 2017) took place over 2 days for each participant. The first day included obtaining informed consent, provision of materials and instructions for biospecimen collection (clean plastic container and OMNIgene.GUT tube (DNA Genotek Inc) for stool and BD Vacutainer urine collection system (Beckton, Dickinson & Co) for urine, at participants’ residences. The next morning, while participants underwent health examinations (at the health center) in a fasting state, fresh morning stool and urine samples collected at home were transferred cooled (~4°C) to the Taabo-Cité central laboratory for immediate pre-analytic processing. In the field, health examinations included point-of-care glucose, hemoglobin, HbA1c, lipids tests, anthropometry (height, weight, neck, arm, waist, and hip circumference), blood pressure, pulse, temperature, as well as preparation of dried blood spots (DBS) on filter papers. At the end of the examination, blood sample (stored at 4°C within 10 minutes of venepuncture) and DBS were sent to the laboratory for further processing. Multimedia Appendix 1 shows the detailed flow of the health examination in the field.

**Figure 3:**
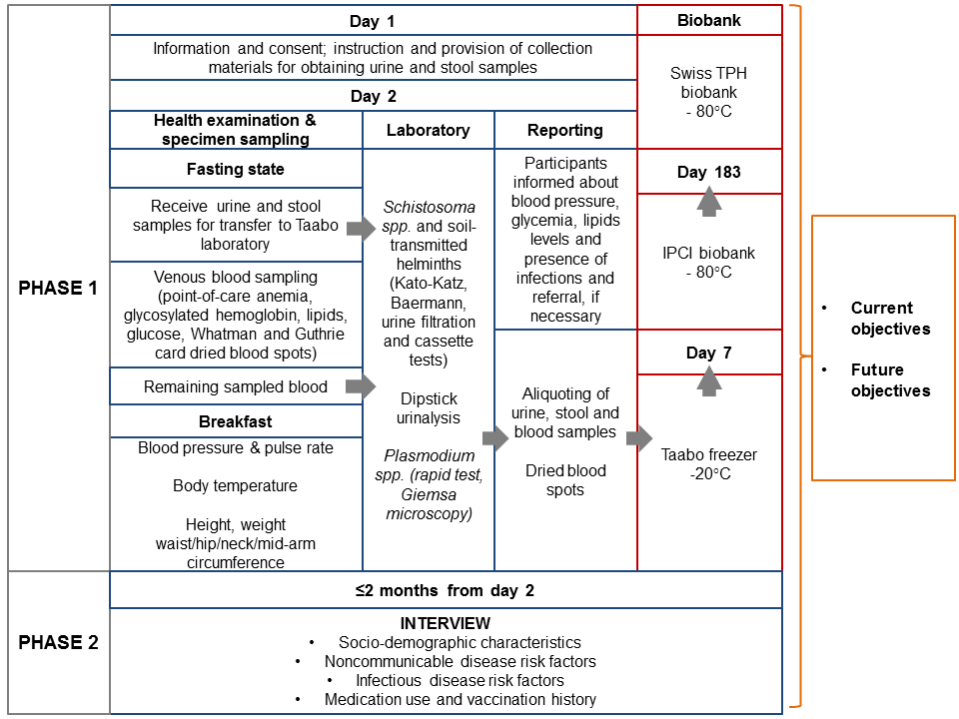
Overall flow of the CoDuBu study (Swiss TPH: Swiss Tropical and Public Health Institute; IPCI: Institut Pasteur de Côte d’Ivoire).

In the laboratory, blood samples were applied towards malaria diagnoses (rapid test and microscopy) and further aliquoted and stored (together with the DBS) in a -80°C biobank. Stool from the plastic container was analyzed for helminth eggs (or larvae), and urine was subjected to *S. mansoni* test and dipstick urinalysis (for blood, proteins, blood, glucose, urobilinogen, bilirubin, leucocytes, ketones, nitrites, pH, and specific gravity). Urine samples that tested positive for blood underwent a filtration method for *S. haematobium*. The remaining urine and OMNIgene.GUT-stabilized stool were aliquoted and transferred to the -80°C biobank. Case reporting and management of diagnosed IDs and NCDs were incorporated within the health examination period (Figure 3). Multimedia Appendix 2 shows the detailed flow of the laboratory work.

In Phase II (interview; June-August 2017), participants were interviewed on a third day, following health examinations. Interviews assessed among other factors, sociodemographic characteristics, health status, ID and NCD risk factors, medication and vaccination history, health care use, as well as knowledge and attitudes regarding NCDs.

Two study teams of about 10 persons each were responsible for the conduct of the study. The teams included 1 research staff (supervision of adherence to study protocol), 1 medical doctor (supervision of physical examination), 3 study nurses (conduct physical examination and point-of-care tests), 1 laboratory supervisor (supervision of work-up and analyses of biospecimens), 1-2 laboratory technicians (work-up and analyses of biospecimens), 2 HDSS field staff speaking the local language(s) and resident in the study area (informed consent, instruction for home biospecimen collection and health interviews), and 1 driver.

### Sample Size Estimation and Sampling of Participants

For the descriptive cross-sectional study, using *Plasmodium* infection and diabetes as our disease co-occurrence of reference, we would expect a malaria prevalence of 30% [59] and diabetes prevalence of 6% [60] in adults. The expected prevalence of co-occurrence assuming independence was 2%. The health district of Tiassalé, where Taabo HDSS is located, has an estimated population of 200,000 [33]. Therefore, for a population of 200,000, with an error margin of 1%, a 95% confidence level, and an expected malaria and diabetes co-occurrence prevalence of 2%, we would need 751 participants. Assuming a nonresponse rate of 30%, the sample size becomes 976. A sample size of 1000 would therefore be enough for the descriptive baseline study.

A main hypothesis to be addressed in an envisaged, not-yet funded follow-up survey, would be that the average change in HbA1c would be higher in persons malaria-positive at baseline. For the power calculation, we assume a malaria positivity rate of 30% and a true difference in average change of HbA1c between malaria-positive and negative persons of at least 30% of the standard deviation (SD) sigma of individual changes. If these individual changes were normally distributed, this would mean that the median of change in malaria-positive persons coincides with the 62^nd^percentile in malaria-negative persons. Under this assumption, 560 persons being re-assessed for HbA1c at follow-up would provide a power of 90% to detect a statistically significant difference in the mean change of HbA1c between the two groups at the usual 5% level. However, a larger sample size is warranted in order to be able to compensate for potential reductions in statistical efficiency resulting from lower than expected malaria positivity prevalence or from variations in the prevalence of malaria-positivity or in the SD sigma across the three study sites. With 30% loss to follow-up, we would still have 700 persons to be re-assessed at follow-up, which would provide a moderate safety margin and allow for the adjustment of weak confounder variables. Therefore, the 1020 participants we sampled from the three selected areas of Taabo HDSS will enable adequate representation of the population and ensure enough statistical power to test our hypotheses.

A random sampling technique was used to select about 2000 participants satisfying the inclusion criteria in the three selected sites. The inclusion criteria included (1) registration in the Taabo HDSS, (2) residence in Taabo-Cité, Amani-Ménou, or Tokohiri, (3) one adult per household, and (4) age ≥18 years. Stratified sampling technique was applied to select 513, 254, and 253 participants from Taabo-Cité, Amani-Ménou, and Tokohiri respectively (ratio of 2:1:1). This corresponds to the overall distribution of adults (Taabo-Cité=3905, Amani-Ménou=2094, Tokohiri=1626) and households (Taabo-Cité=1508, Amani-Ménou=567, Tokohiri=616) in the three areas. Selected participants from each stratum were then invited for participation in a random fashion until the target sample size in each site was reached.

### Recruitment of Participants and Written Informed Consent

After an information event for the village population, informed consent from potential participants was obtained at their homes, in-person by HDSS field workers who are in close contact with the local population. In order to obtain written informed consent, the field workers explained the purpose, procedures, benefits, and potential harm of the study. The concept of establishing a biobank for future studies was also explained, and participants were made aware that their information would be treated in a coded manner to allow the establishment of a longitudinal study if they decided to participate. The voluntary nature of the study was explained to the participants. Participation in all or parts of the study, as well as nonparticipation was free of negative consequences. Sufficient time for asking questions on the aims and procedures of the study was provided to the participants. If a subject decided to participate, they signed the informed consent sheet. Illiterate participants put a cross in the presence of a literate witness. Consent for re-contact in the future was specifically obtained from all participants towards the establishment of a longitudinal cohort.

### Assessment of Health-Related Phenotypes

Textbox 1 summarizes the health-related phenotypes expected from the study. These phenotypes will be derived from physical examinations; laboratory analyses of venous blood, urine, and stool; and in-person interviews with a questionnaire.

### Physical Examination

Body temperature was measured with an Omron auricular thermometer (Omron Healthcare). Systolic and diastolic blood pressure were measured three times, at least 3 minutes apart, after sitting quietly for about 10 minutes, using an Omron Oscillograph (Omron Healthcare). The mean of the last two measurements will be taken for analyses. Hypertension will be defined as a mean systolic blood pressure ≥140 mm Hg or mean diastolic blood pressure ≥90 mm Hg.

Height and weight were measured using a SECA bodymeter (0.1 cm accuracy) and weighing scale (0.1 kg accuracy), respectively. Precision of the bodymeter was checked regularly by measuring the height of the placement of the bodymeter using a tape rule, and the weighing scale was verified using a known 10 kg weight. Neck circumference was measured using a SECA ergonomic tape below the laryngeal prominence in front and the mid-cervical spine behind. Mid-arm circumference was measured at the midpoint between the shoulder and the elbow. Waist circumference was measured at the end of passive expiration over the narrowest part of the trunk between the lowest rib and the iliac crest, or if this is not clearly evident, at the level of the umbilicus. Hip circumference was measured using the same tape, at the maximal circumference between the iliac crest and the crotch. Waist circumference, waist-hip ratio, and body mass index will be described in absolute values and categorized according to various guidelines [61].

### Laboratory Screening

#### Venous Blood

A total of 7 mL of venous blood was collected in a fasting state for laboratory screening and storage in biobank. Five mL of venous blood (collected using an ethylenediaminetetraacetic acid [EDTA] tube) was used for the assessment of glucose, HbA1c, lipids, as well as for assessment of *Plasmodium*, and anemia. HemoCue Glucose 201 RT device was used to measure fasting glucose (HemoCue AB) and was calibrated regularly. Afinion AS100 Analyzer (Alere GmbH) was used as the point-of-care test for HbA1c (where the method has been validated for HbA1c testing in settings with high prevalence of hemoglobinopathies) and lipid profile (total cholesterol, low-density lipoprotein cholesterol, high-density lipoprotein cholesterol, and triglycerides). Afinion test kits were stored at 4°C, with regular calibration of the analyzers. For assessment of *Plasmodium* infection, thick and thin blood films were prepared on microscope slides. For the diagnosis of *Plasmodium*, the slides were air-dried, stained with Giemsa, and examined under a microscope by experienced laboratory technicians for *Plasmodium* species identification and parasitemia. The number of parasites was counted against 200 leukocytes or 500 leukocytes if number of parasites was ˂10, assuming a standard count of 8000 leukocytes per μl of blood [62]. Additionally, a drop of blood was subjected to a rapid diagnostic test for malaria using ICT ML01 malaria Pf kit (ICT Diagnostics). Anemia was indirectly assessed by the hemoglobin level, using HemoCue Hb 301 device (HemoCue AB), adhering to World Health Organization recommendations [63]. The remaining EDTA-buffered blood was processed into the biobank.

Two mL of venous blood (collected using a dry tube) was used to prepare DBS on custom printed diagnostic cellulose filter paper (Whatman) for DNA methylation profile and neonatal Guthrie cards for cytokine profiles.

**Key health-related phenotypes in the CoDuBu study investigating the co-occurrence of common infectious diseases and noncommunicable diseases in Côte d’Ivoire.**

**A. Key phenotypes from the physical health examination:**

1. Distribution of body temperature (auricular temperature)
2. Distribution of height, weight, waist and hip circumference, prevalence of obesity
3. Distribution of blood pressure and heart rate and prevalence of hypertension

**B. Key phenotypes from the laboratory screening:**

1. Prevalence of *Plasmodium* infection
2. Prevalence of urogenital schistosomiasis (*Schistosoma haematobium*)
3. Prevalence of intestinal schistosomiasis (*S. mansoni*)
4. Prevalence of species-specific soil-transmitted helminths (*Ascaris lumbricoides*, hookworm and *Trichuris trichiura*)
5. Prevalence of *Strongyloides stercoralis*
6. Prevalence of anemia
7. Prevalence of prediabetes/diabetes
8. Prevalence of dyslipidemia

**C. Key phenotypes from interview (>200 variables covering risk factors and effect modifiers):**

1. Prevalence of doctor’s diagnoses/treatments, including HIV, tuberculosis
2. Prevalence of health symptoms related to cardiovascular diseases, diabetes, respiratory diseases, and infectious diseases
3. Distribution of access to quality care
4. Distribution of knowledge related to noncommunicable diseases and risk factors
5. Prevalence/exposure to noncommunicable disease risk factors: tobacco smoking, physical activity, nutrition, alcohol consumption, environmental exposures (air pollution from fossil fuels, occupational exposures, noise annoyance), gender/reproductive factors, stress, resilience, depression, and other psychosocial factors

**D. Other variables derived from the Taabo health and demographic surveillance system database:**

1. Sociodemographic factors (age, sex, nationality, ethnicity, marital status, pregnancy history, educational level, occupation, socioeconomic status assessed by a household-based asset index)
2. Household characterization (size, water supply, sanitation and cooking facilities, building characteristics, and residential history)

**E. Biobank:**

1. Dried blood spots/aliquots from venous blood: genetic variation, DNA methylation, cytokine profile
2. Stool: gut microbiome
3. Urine: metabolome

#### Urine Sample

A midstream morning urine sample was collected using BD Vacutainer system (Beckton, Dickinson & Co) and transferred to the laboratory for processing. Nine mL of urine was taken from the vacutainer using a separate vacuette, which was later processed into the biobank. A small amount (10-20 µl) of urine was used for the detection of *S. mansoni*, using the point-of-care circulating cathodic antigen cassette (ICT Diagnostics), following the manufacturer’s instructions [64], resulting in the following classification: negative or three levels of positives (1+, 2+, or 3+). Dipstick urinalysis to detect proteins, blood, glucose, urobilinogen, bilirubin, leucocytes, ketones, nitrites, pH, and specific gravity was applied using the Roche Combur-10 test (Roche Diagnostics). Urine samples testing positive for blood were subjected to filtration test for the diagnosis of *S. haematobium.* Positive results were expressed as the number of *S. haematobium* eggs per 10 mL of urine, according to World Health Organization guidelines [65].

#### Stool Samples

Fresh morning stool samples were collected into plastic containers with a volume of 125 mL and tightly fitting lids. Samples were transferred to the laboratory in a cool box and worked up the same day. For the diagnosis of *S. mansoni* and soil-transmitted helminths, duplicate 41.7 mg Kato-Katz thick smears were prepared from each stool sample [22] and examined under a microscope. Infection intensities were expressed in eggs per gram of stool. For the diagnosis of *S. stercoralis*, the Baermann technique was employed, where an apricot-sized stool sample was placed on a gauze-lined mesh in a glass funnel equipped with a rubber tube and a clamp, covered with deionized water, and illuminated from below with a bulb. After 2-3 hours, the lowest 50 mL of the liquid was drained, centrifuged, and the sediment examined under a microscope for *S. stercoralis* larvae [22]. A separate stool sample was also collected in a self-administered manner by the participants (following the manufacturer’s instructions) using the OMNIgene.GUT tube, which contains microbial stabilizer.

### Pre-Analytic Processing of Biospecimens and Biobanking

DBS prepared on filter papers were sealed in air-tight bags containing dessicants and stored at -20°C in the Taabo laboratory. The DBS will be applied towards DNA and cytokine profiles. The DNA that will be extracted from the DBS (Whatman FTA cards) will facilitate research into genetic variations and DNA methylation [50]. EDTA blood was stored as 1.5 mL aliquots in cryotubes at -20°C in Taabo laboratory. Midstream morning urine specimens collected into sterile 9 mL vacuettes were stored as uncentrifuged and centrifuged 1.5 mL urine aliquots. Centrifugation was done at 1600 g and 4°C for 15 min [66], and the supernatant immediately aliquoted in cryotubes stored at -20°C in Taabo laboratory. Urine aliquots will be applied to metabolomics profiles and will be normalized to creatinine during the analytic process. Stool samples from the OMNIgene.GUT tube were stored in 0.5 mL aliquots in cryotubes at -20°C freezer in Taabo laboratory. These stool aliquots will be applied towards the metagenomic investigations of the gut microbiota.

DBS, blood, urine, and stool samples stored at -20°C in the Taabo laboratory (with the freezer being connected to a back-up generator) were transferred (on dry ice, on a weekly basis) to the Institut Pasteur de Côte d’Ivoire (IPCI) biobank for permanent storage at -80°C (Figure 3). On completion of the survey and therefore of biospecimen collection, the biobank will be mirrored as two identical banks hosted at IPCI (Abidjan, Côte d’Ivoire) and the Swiss Tropical and Public Health Institute (Swiss TPH; Basel, Switzerland). The mirroring of biobanks increases the safety of the biospecimens. Biomarkers derived from these “-omics” analyses can serve as exposure biomarkers, health-related phenotypes, or markers of susceptibility of exposures and diseases.

### In-Person Interviews

The in-person interviews were done using a tablet-based questionnaire in Open Data Kit format [67], lasting between 45 and 60 minutes. Questions covered demographic factors (eg, age, sex), water supply and sanitation facilities (availability and use), know-how, attitude, and behavior towards tobacco smoking, physical activity, obesity, alcohol consumption, and nutrition. They also included environmental risks (eg, exposure to mosquito coils, pesticides, indoor biomass fuels), gender, hormonal and reproductive factors, psychosocial stressors and resilience to them, disease symptoms (respiratory, cardiovascular, and diabetes), family history of disease, medical diagnoses and treatments, vaccination history, and access to health care.

### Data Management

Data from in-person interviews were collected using Open Data Kit, whereas health examination and laboratory data were collected as paper-based records, which are later uploaded into the Open Data Kit format. Data quality was assured through (1) formulation of standard operating procedures for all aspects of the study, (2) extensive and careful training of the study team according to the standard operating procedures, (3) onsite supervision of field activities ensuring adherence to protocol, and (4) continuous monitoring and internal evaluation of data entry during the field and laboratory work. Data collected on paper will be double-entered in two stages and later cross-checked to ensure accuracy and prevent data manipulation during or after the study. The software used will keep track of all changes made to the data. All data will be merged into a single database at the end of data entry using STATA version 14.0 (STATA Corporation).

### Data Analyses

The detailed CoDuBu data, enabled in part by HDSS routine data collected just before the start of this survey (Textbox 1), and the broad consent obtained from participants will allow us to test numerous hypotheses. The broad range of the CoDuBu research framework translates into a broad set of statistical methods. Analytical study plans or project proposals will be developed and submitted to statistical review at participating organizations. Collected data will include continuous and categorical variables. Results from the cross-sectional study will be described accordingly, reporting categorical variables as percentages with 95% confidence intervals, and continuous variables as medians with interquartile ranges. To explore associations between predictors and health outcomes, linear or logistic regression models will be used, as appropriate. Urban and rural differences in associations will be tested, as well as fixed and random effects study area models. Sampling weights will be applied to analyses to ensure a correct representation of the population. Missing data will be reported as separate categories and included in multivariable models. If full case analyses are performed, adjustment for data missingness will be done using appropriate methods for missing data imputation.

### Ethical Approval and Data Protection

Ethical approvals for the study were obtained from the Ethics Committee Northwest and Central Switzerland (reference no. 2016-00143; obtained May 2, 2016) and the Côte d’Ivoire National Research Ethics Committee (reference no. IORG00075; obtained March 24, 2017). Data entry will be done using password-protected tablet computers. Only participant identifiers, but not names of participants will be included in electronic health databases. Paper-based records from the laboratory and filled-in forms such as biospecimen-collection forms also contain only the participant identifier and are kept in a locked cupboard in a room with access restricted to the project personnel. The CoDuBu data will be handled only by authorized staff at the CoDuBu partner organizations.

After completion of the data entry/cleaning, identical databases for the CoDuBu project will be stored at Centre Suisse de Recherches Scientifiques en Côte d’Ivoire (CSRS) in Abidjan, Côte d’Ivoire, and Swiss TPH in Basel, Switzerland. Access to data in the context of a project application will need approval from the bipartite project leaders. Collaborating researchers will receive datasets for analyses that are coded only with the participant identifier, stripping any other identifying information (eg, name, birth date) from the dataset. Data transfer agreements will be signed with external scientific collaborators.

Biospecimen aliquots were barcode-labeled and doubly coded (a separate biospecimen identifier that is different from the participant identifier), improving confidentiality of participants. At the IPCI and Swiss TPH biobanking facilities, freezer temperatures are supervised and recorded continuously by two independent temperature control systems that assure biospecimen safety. Access to freezers and biospecimens are restricted to key study personnel. Access to biospecimens for biomarker analyses in the context of a project will need approval from the project leaders. The principal investigators will prioritize the future relevant research questions and coordinate submission of future research projects to the ethics committees in Côte d’Ivoire and Switzerland. Biospecimen transfer agreements will also be signed with external scientific collaborators.

Generation of the identifiers was done systematically. The participant identifier consists of six characters where the first two are the site number (10, 11, and 12 for the three sites) and the last four characters are the participant’s serial number within the study site (starting from 0001). For instance, the first participant from site 10 was assigned a participant identifier of 100001. The biospecimen identifier comprises nine characters: a different site identifier (21, 31, 41) as well as different within-site serial numbers (starting from 0247), and biospecimen-specific suffices (uncentrifuged and centrifuged urine and stool). Thus, the first stool aliquot from the first participant in site 21 was assigned 210247STL1. A file linking the participant identifier to the biospecimen identifier and finally to the existing unique Taabo HDSS identifier was created and kept only by the co-principal investigators of the CoDuBu project.

All data generation, management, storage and analyses, as well as the storage and management of biological samples, strictly follow the Swiss and Côte d’Ivoire legal requirements for data protection.

### Health Systems Preparation and Diagnostic Follow-Up

At the end of the data collection for this baseline survey, participants were informed on IDs, NCDs, and their risk factors in order to promote healthy lifestyle. Information and education campaigns—through radio, posters, and flyers—were established for education of the population. Participating health centers were equipped with facilities for hypertension and diabetes screening, and the main hospital at Taabo-Cité was made a referral central center for initial NCD diagnosis and treatment in the area. Medical staff on the project were trained and certified for performing electrocardiography. Participants who were diagnosed with any screened infection were treated free of charge. Participants diagnosed with any NCD were followed up for confirmation. Transportation fees for confirmation and first month of treatment were covered by the study. Further management are being co-ordinated by study partners in Côte d’Ivoire, ensuring patient enrollment in a subsidized treatment plan.

## Results

In September 2017, we had completed participant recruitment and questionnaire administration. In parallel to the questionnaire administration, data cleaning and processing of the health examination results were employed and should be completed by the end of October 2017. We expect the preliminary results by December 2017. Study findings will be published in peer-reviewed literature and presented at national and international conferences.

## Discussion

### Principal Considerations

Results from this comprehensive baseline survey will provide an overall insight into the relationship between IDs and NCDs in a primarily rural setting of Côte d’Ivoire that is undergoing rapid epidemiological transition. The understanding of potential mutual influences between IDs and NCDs is relevant because their co-occurrence at an individual level will require the adaptation of health service provision towards integrated care. If IDs and NCDs influence each other (and their risk factors) in light of the increasing prevalence of NCDs, country-specific risk estimates will be needed to increase the precision of local disease burden towards a deeper understanding of global burden of disease. In addition, if IDs increase NCD susceptibility and induce a shift towards a younger age of onset, this would also require adaptation in the target population for NCD screening in LMICs and country-specific contexts. Research into the dual disease burden, particularly in the context of biobanks and biomarkers, will improve the mechanistic understanding of the ID-NCD relationship and refine health impact assessment of ID and NCD prevention and control programs. The preparation of the health system will improve awareness of NCD risk factors, educate local health staff on disease management, and create a referral center in an area that, thus far, had limited expertise, necessitating lengthy travel to receive adequate care.

The integration of this project into the Taabo HDSS made the execution of the baseline study quite cost-effective. Apart from providing a recruitment base as well as sociodemographic information of the population, the HDSS made it easier to recruit individuals given their familiarity with research and confidence in the system. In addition, the system provides a low risk for loss to follow-up since their sustainable field work teams hold close contact with the local population. In case of loss to follow-up, we would have the reasons and address them accordingly.

A pilot survey served to identify bottlenecks, which were addressed prior to launching this comprehensive baseline survey. We generally did not have much difficulty in undertaking the baseline study. Although the study area is mostly rural with a relatively lower literacy level and considerable poverty, the awareness for health research is high. This is explained by health research and interventions dating back to the late 1990s [68] and the establishment and running of the Taabo HDSS since 2009, including specific health surveys, large-scale NTD control interventions, and strengthening the health system [7,33,54]. Since most of the previous studies in the area have been on children [56-59,69], the village chiefs were pleased that our study would focus on adults and would be the first one to investigate cardiovascular and metabolic diseases.

The project partners have the necessary collective expertise in ensuring a successful execution of the project [49,68-73]. The current project was implemented in close collaboration between CSRS, Swiss TPH, IPCI, Institut National de Santé Publique, Ligue Ivoirienne contre l’Hypertension Artérielle et les Maladies Cardiovasculaires, and Université Félix Houphouët-Boigny. CSRS is one of the leading centers in Africa in integrated research on health, environment, and nutrition. The Taabo HDSS is a major resource center of CSRS, and several multiyear research projects have been jointly implemented in the Taabo HDSS and have generated high-quality data regarding the etiology of anemia, the epidemiology of malaria, and integrated control of zoonoses and NTDs [54-59,69], which have contributed to national policies regarding these diseases. Swiss TPH has a track record in IDs and NCDs research, which forms the basis for the scientific objectives of this project. Swiss TPH is running the only Swiss-wide chronic disease cohort and biobank (Swiss Cohort Study on Air Pollution and Lung and Heart Diseases in Adults), participates at the forefront of exposome research [49,71,74], and its coordinators have vast experience in biobanking (Swiss Biobanking Platform). The Université Félix Houphouët-Boigny is the largest public university in Côte d’Ivoire with several laboratories covering ecology, parasitology, and zoology. Institut National de Santé Publique coordinates management of diabetes on a national level, while Ligue Ivoirienne contre l’Hypertension Artérielle et les Maladies Cardiovasculaires coordinates the efforts at public health control of cardiovascular disease at the national level.

There is an existing team of 30 experienced staff operating the Taabo HDSS, including field workers, data managers, and administrative staff [33]. Local physicians and nurses will also contribute to the manpower needed for the overall execution of this project. Expertise in biobanking available from Swiss TPH, CSRS, and IPCI (responsible for the Côte d’Ivoire national biobank) will ensure effective sample preservation, handling, and processing during the project and beyond.

### Limitations

Despite the novelty of our research focus, we expect some limitations. First, our sample size is relatively limited compared to other NCD cohorts. Although this limitation is mainly related to funding, our estimation shows that we would have enough statistical power to detect relevant trends and associations. We also do not expect a lot of loss to follow-up as all participants willingly accepted to be re-contacted in due course. The success of the baseline survey will lead to application for competitively acquired funds towards expansion in planned follow-up surveys, both population-wise and health endpoint-wise, giving a clearer picture of initially observed trends and associations. We could not perform HIV and tuberculosis testing due to logistic/cultural reasons. Both conditions are dependent on immunologic pathways, and their related markers could modify or mediate ID-NCD relationships. However, questions about these conditions are covered in the questionnaire and we would therefore rely on self-reported history or use of medications for these infections in the baseline cross-sectional investigations. Efforts will be made to include these tests in the follow-up survey.

### Conclusion

The CoDuBu study will fill the void of lacking data in areas of potential links between IDs and NCDs and shed new light on respective risk factors. Our findings will lead to the development of integrated approaches, which hold promise for cost-effective prevention and management of dual disease burden. Future research from established research infrastructure will contribute to local capacity building, deeper understanding of the cross-talk between IDs and NCDs and treatment, and precise country-specific burden of disease and risk factor estimates to guide policy.

## Appendix

Multimedia Appendix 1 Detailed flowchart of field activities for the CoDuBu study.

Multimedia Appendix 2 Flow of laboratory tests and biobanking in the CoDuBu study.

## Abbreviations

CoDuBu: Côte d’Ivoire Dual Burden of Disease study
CSRS: Centre Suisse de Recherches Scientifiques en Côte d’Ivoire
DBS: dried blood spot
HbA1c: glycosylated hemoglobin
HDSS: health and demographic surveillance system
HIV: human immunodeficiency virus
ID: infectious disease
IPCI: Institut Pasteur de Côte d’Ivoire
LMICs: low- and middle-income countries
NCD: noncommunicable disease
NTD: neglected tropical disease
Swiss TPH: Swiss Tropical and Public Health Institute
