# Supplementary material for: Côte d’Ivoire Dual Burden of Disease (CoDuBu): Study Protocol to Investigate the Co-occurrence of Chronic Infections and Noncommunicable Diseases in Rural Settings of Epidemiological Transition
Source: JMIR Res Protoc. 2017 Oct 27;6(10):e210. doi: 10.2196/resprot.8599 (PMC5681722; doi:10.2196/resprot.8599)
Supplement: Multimedia Appendix 1 [file resprot_v6i10e210_app1.pdf]

Day 1 (18.00-23.00): Participants' residence

- Identification of potential participants (~30 per day) based on sampling frame provided by the Taabo HDSS
- Explanation of the study
- Distribution of stool, urine and OMNIgene.GUT containers in a styrofoam box containing ice packs
- Instruction on biospecimen collection and next-day health centre visit

Day 2: Health centre: (06.00-11.00)

1. Registration and identification (~30 minutes)

- Arrival of participant at the health centre, in a fasting state, with the Styrofoam box containing biospecimens.
- Signing of consent form; assignment of identifier to participant and labelling of the biospecimens and all study materials to be used by the participant (PID).
- Biospecimens transferred in a central cooling box containing ice packs for later transfer to the laboratory

2. Venepuncture (~5 minutes): 2 ml dry tube and 5 ml EDTA tube.

3. Breakfast (~30 minutes)

4. Physical examination (~15 mins)

- Blood pressure and pulse (3 times; at least 3 minutes apart)
- Height, weight, neck, arm, waist and hip circumference
- Body temperature
- All results noted in the data sheet

5. Consultation (~30 mins)

- Participant gets a consultation with the medical doctor (based on examination results) on site for diagnosis
- Counselling, treatment or referral as required.
- If diabetic, hypertensive, or tachycardic, ECG is done to assess cardiac function and further action taken if necessary

Venous blood handling

- First, 2 ml blood (from dry tube) for staining Whatmann FTA and Guthrie cards (~5 mins); cards left to air-dry (~2 hrs).
- Then, 1-2 ml from 5 ml EDTA blood used for glucose, haemoglobin, HbA1c and lipids tests in that order. Results noted in the data sheet. (~15 mins)
- Remaining EDTA blood put in a Styrofoam box containing ice packs for later transfer to the laboratory
- Dried blood spots sealed in air-tight bags containing desiccants and also transferred to the laboratory for storage

4. Transfer to the laboratory

- Central cooling box containing biospecimens - stool, urine, OMNIgene.GUT samples (on ice packs, at ~9.00)
- Styrofoam box containing 5ml EDTA tube containing ~3 ml blood (on ice pack; at ~10.00)
- Plastic box containing all dried blood spots sealed in air-tight bags containing desiccants (at ~10.00)
