# Supplementary material for: Côte d’Ivoire Dual Burden of Disease (CoDuBu): Study Protocol to Investigate the Co-occurrence of Chronic Infections and Noncommunicable Diseases in Rural Settings of Epidemiological Transition
Source: JMIR Res Protoc. 2017 Oct 27;6(10):e210. doi: 10.2196/resprot.8599 (PMC5681722; doi:10.2196/resprot.8599)
Supplement: Multimedia Appendix 2 [file resprot_v6i10e210_app2.pdf]

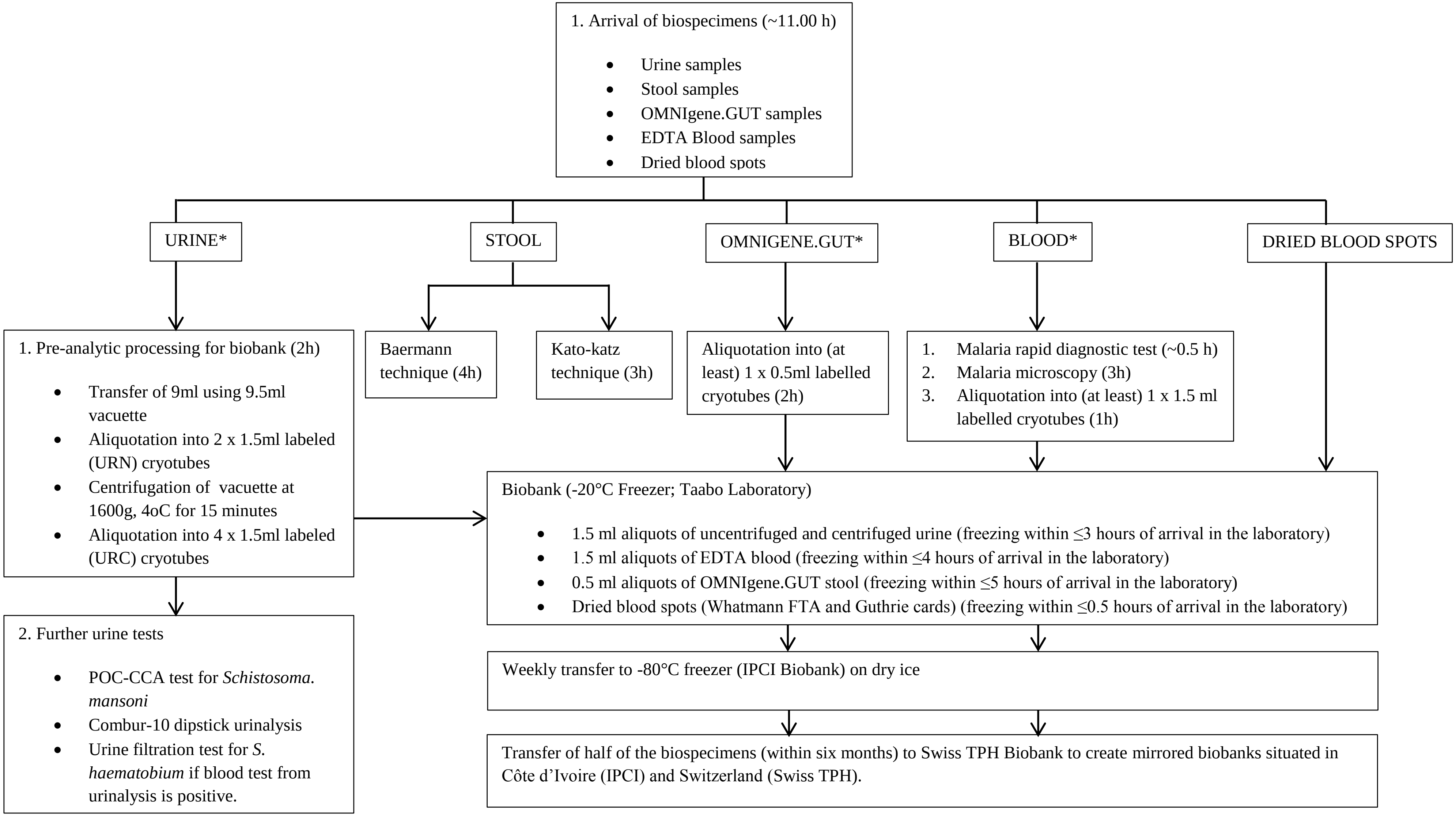

*Biospecimen kept in refridgerator until analyses and aliquoting. IPCI: Institut Pasteur de Côte d,Ivoire; Swiss TPH: Swiss Tropical and Public Health Institute
